# FOXP1 regulation via the PI3K/Akt/p70S6K signaling pathway in breast cancer cells

**DOI:** 10.3892/ol.2015.2885

**Published:** 2015-01-16

**Authors:** SEVIL OSKAY HALACLI, AYSE LALE DOGAN

**Affiliations:** 1 Pediatric Immunology Unit, Institute of Children’s Health, Hacettepe University, Ankara 06100, Turkey; 2 Department of Basic Oncology, Institute of Oncology, Hacettepe University, Ankara 06100, Turkey

**Keywords:** phosphatidylinositol 3-kinase, Akt, p70S6 kinase, Forkhead box P1, breast cancer

## Abstract

Loss of Forkhead box P1 (FOXP1) protein expression confers a poor prognosis in sporadic and familial breast cancer patients, and the *FOXP1* gene maps to a tumor suppressor locus at chromosome 3p14. Although correlation studies have indicated that FOXP1 has a role in tumor suppression, determination of the regulatory mechanism of FOXP1 is required to establish its function in breast cancer. It has previously been identified that FOXP1 is regulated by estrogen in breast cancer and that treatment with bisphenol A is effective for regulating the transformation of the normal human breast epithelial cell line, MCF-10F. In addition, FOXO-regulated activation of FOXP1 inhibits the apoptosis of MCF-10F cells following tamoxifen and Akt inhibitor VIII administration. The present study indicates that FOXP1 regulation occurs via a PI3K/Akt/p70S6 kinase (p70S6K) signaling pathway. Following treatment with wortmannin, an inhibitor of phosphatidylinositol 3-kinase (PI3K)/Akt, MCF7 and MDA-MB-231 breast cancer cells demonstrated decreased FOXP1 protein expression levels; this result was also observed in the small interfering (si)RNA silencing of Akt. By contrast, overexpression of Akt resulted in increased FOXP1 protein expression levels in the MDA-MB-231 cells compared with the control cell lysates. Furthermore, treatment with rapamycin, a specific inhibitor of the mammalian target of rapamycin/p70S6K cascade, resulted in decreased FOXP1 expression in the MCF7 cells, but not in the MDA-MB-231 cells, which were resistant to rapamycin-induced inhibition. In addition, silencing of p70S6K using siRNA produced a marked decrease in FOXP1 expression. These data indicate that FOXP1 protein expression is regulated by a PI3K/Akt/p70S6K signaling cascade in breast cancer.

## Introduction

The phosphatidylinositol 3-kinase (PI3K)/Akt signaling pathway is associated with variable cellular functions critical to tumor initiation and progression, including proliferation, migration, invasion and metastasis, as well as the acquired endocrine resistance of breast cancer following hormonal therapy (1,2). Akt, an important downstream Ser/Thr kinase, performs its functions by activating or inactivating ~200 different downstream targets (3). One of these downstream targets is p70S6K, a Ser/Thr kinase that is regulated by PI3K/Akt. The roles of p70S6K are not limited to protein translation, but additionally involve the co-regulation of other cellular responses, such as cell survival, proliferation and migration (4–6).

Forkhead box (FOX) transcription factors are another group of proteins regulated by PI3K/Akt. Akt negatively regulates FOXO transcription factors by promoting proteosomal degradation (7) and the nuclear exclusion of FOXO proteins (7–9). Furthermore, van Boxtel *et al* (10) demonstrated that the therapeutic targeting of PI3K/Akt with tamoxifen facilitates the nuclear import of FOXO, inducing FOXP1 protein expression (10).

FOXP1, a member of the winged helix transcription factors, was initially described by Banham *et al* (11), using the novel JC12 antibody to locate the *FOXP1* gene at a tumor suppressor locus on chromosome 3. Although FOXP1 mRNA and protein expression was identified in a wide range of health human tissues (11), the subcellular localization of the FOXP1 protein varied between different tissues. For example, nuclear FOXP1 expression was predominantly identified in tissues such as the kidney, thyroid, cerebellum, tonsils, blood and colon, whereas cytoplasmic expression was predominantly observed in epithelial tissues such as the stomach, colon and lung macrophages (11). Similarly, FOXP1 protein expression levels and localization vary depending on the tissue type and disease stages in cancer. For example, strong nuclear staining and/or heterogeneous weak nuclear staining of FOXP1 were identified in different stages of breast cancer progression (11–13).

Functionally, FOXP1 protein expression has opposing effects in different types of cancer. In tumors such as diffuse large B-cell lymphoma, glioblastoma and hepatocellular carcinoma, FOXP1 exhibits oncogenic functions, however, in breast cancer, FOXP1 has been detected to act as a tumor suppressor (11,12). Thus, loss of nuclear FOXP1 expression is correlated with a poor prognosis in breast cancer. Previously, increased FOXP1 expression demonstrated a significant positive association with estrogen receptor-α (ER-α) expression in the relapse-free, borderline and overall survival of primary human breast carcinoma patients (13). Furthermore, nuclear FOXP1 expression in primary invasive breast carcinoma was positively correlated with nuclear ER-β expression (14); and this correlation was associated with a low tumor grade and high survival in primary invasive (13) and familial breast cancer. Although a number of correlation studies have demonstrated an association between clinical outcome and FOXP1 protein expression, only a limited number of studies have been conducted to investigate the function of FOXP1 in breast cancer (10,15). Rayoo *et al* (16) identified increased FOXP1 mRNA expression in MCF7 breast cancer cells following estrogen treatment, with elevated FOXP1 protein levels causing an increase in MCF7 cell proliferation. Furthermore, FOXP1 appeared to increase transcription driven by the estrogen response element, and in relapse-free patients treated with tamoxifen, FOXP1 immunoreactivity was significantly increased (16). By contrast, *FOXP1* hypermethylation was found following bisphenol A (BPA) exposure in MCF10F healthy immortal breast cells. Although FOXP1 does not appear to have a binding site for Akt, treatment with Akt inhibitor VIII at various time-points resulted in increased FOXP1 expression levels (10). This may be due to FOXP1 exhibiting recognition sites for p70S6K, a downstream molecule of the PI3K/Akt cascade (11). Considering the aforementioned findings, the aim of the present study was to investigate the regulation of FOXP1 via the PI3K/Akt/p70S6K signaling cascade in breast cancer cells.

## Materials and methods

### Cell culture, and treatment with growth factors and pharmacological inhibitors

Commercially available MCF7, ZR-75.1, and MDA-MB-231 cancer cell lines were provided by Professor Alison H. Banham (Nuffield Department of Clinical Laboratory Sciences, University of Oxford, Oxford, UK) and grown in Dulbecco’s modified Eagle’s medium (DMEM; Fisher Scientific International Inc., Hampton, NH, USA) supplemented with 10% fetal bovine serum (Fisher Scientific International Inc.), 100 μg/ml penicillin/streptomycin (Fisher Scientific International Inc.) and 2 mmol/l L-glutamine (Fisher Scientific International Inc.). The cells were plated at a density of 1×10^6^ cells per well on a 6-well plate and placed in a humidified incubator at 37°C containing 5% CO_2_ (Fisher Scientific International Inc.) overnight. Following serum starvation for 24 h, the cells were incubated with human recombinant epidermal growth factor (EGF; Invitrogen Life Technologies, Carlsbad, CA, USA) at a final concentration of 50 ng/ml for 20 min. Wortmannin and rapamycin (Cell Signaling Technology, Inc., Danvers, MA, USA) were added to the cells 15 min prior to growth factor stimulation at final concentration of 100 nM.

### Antibodies

Anti-phospho-Akt, anti-Akt, anti-phospho-p70S6K and anti-hemagglutinin (HA) antibodies were purchased from Cell Signaling Technology, Inc., and used at a dilution of 1:1,000, according to the manufacturer’s instructions. The JC12 anti-FOXP1 antibody was provided by Professor Alison H. Banham and used at a dilution of 1:30. β-actin mouse monoclonal antibody (dilution, 1:5,000) and horseradish peroxidase-conjugated anti-mouse antibody (dilution, 1:2,500) were purchased from Sigma-Aldrich (St. Louis, MO, USA) and Agilent Technologies, Inc. (Santa Clara, CA, USA), respectively.

### Plasmids and RNA interference

1071 pBabe puroL myrAkt T308A S473A (plasmid no. 9014), 1072 pBabe puroL myrAkt K179M T308A S473A (plasmid no. 9015)and PBABE empty vector (plasmid no. 21836) were obtained from Addgene, Inc. (Cambridge, MA, USA), and pAB195-FOXP1 expression plasmid and green fluorescent protein-tagged pAB195 vector expressing full length FOXP1 were provided by Dr Philip J. Brown (University of Oxford). Additionally, a SignalSilence^®^ Akt small interfering (si)RNA kit (cat no. 6511), p70/85 S6 kinase (S6K) siRNA I (cat no. 6566), p70/85 S6K siRNA II (cat no. 6572) and control siRNA (cat no. 6568) were purchased from Cell Signaling Technology, Inc.

### Plasmid digestion

Plasmid DNA (500 ng) was digested using *Hin*dIII and *Bam*HI restriction enzymes (New England BioLabs, Inc., Ipswich, MA, USA) at 37°C in an agitated water-bath for 1 h. Subsequently, the digested plasmids were evaluated on 1% agarose gel (Sigma-Aldrich).

### Transfection

TransPass™ D1 transfection reagent (New England BioLabs, Inc.) was used for plasmid transfection. The MDA-MB-231 cells were seeded at a density of 1×10^5^ cells per well on a 24-well plate, and incubated with 1 μg plasmid DNA mixed with 3 μl transfection reagent in serum-free high glucose DMEM (Lonza, Basel, Switzerland) at room temperature for 30 min. The cells were washed twice with serum-free DMEM prior to replacing the DMEM with transfection mixture. The cells were then incubated for 5 h priot to the mixture being replaced with complete growth medium for 48 h. TransPass R2 siRNA transfection reagent (New England Biolabs, Inc.) was applied to conduct siRNA transfection. The cells (1×10^5^ cells per well) were plated in growth medium without antibiotic on a 12-well plate, in a humidified incubator at 37°C containing 5% CO_2,_ overnight. The transfection mixture was produced by mixing 1.25 μl TransPass R2 solution A to 600 μl serum-free high glucose DMEM, then adding 2.5 μl Transpass R2 solution B and mixing well. Finally, 40 nM siRNA was added to the transfection mixture, which was incubated at room temperature for 20 min. The cells were washed twice with serum-free DMEM prior to replacing the DMEM with the transfection mixture, and then incubated for 4 h prior to adding 1 ml growth medium. After 48 h of transfection, the cells were lysed and western blot analysis was performed.

### Western blot analysis

The cells were lysed using 1× cell lysis buffer (Cell Signaling Technology, Inc.), including 1 mM phenylmethylsulfonyl fluoride, and transparent protein lysates were obtained following centrifugation at 29,994 × g. Bradford assay reagent (Fisher Scientific International Inc.) was used for protein quantitation, and 40-μg protein samples were separated using 5% stacking and 10% resolving gels at 60 V for 1 or 2 h. The separated proteins were immunoblotted to a polyvinylidene difluoride membrane using iBlot^®^ dry blotting system (Invitrogen Life Technologies) and blocked for 1 h with 5 % skimmed milk in Tris-buffered saline and 1% Tween 20 (Sigma-Aldrich). Protein bands were visualized with the SuperSignal West Femto Chemiluminescence kit (Fisher Scientific International Inc.) and analyzed using Kodak Gel Logic 1500 imaging system software (Eastman Kodak Co., Rochester, NY, USA). For all experiments, a minimum of three independent experiments were performed and representative results were selected for analysis.

### Statistical analysis

Statistical analyses were performed using GraphPad Prism 5.0 package (GraphPad Software Inc., La Jolla, CA, USA*)*. Student’s t-test was used to statistically analyze the expression differences. P<0.05 was considered to indicate a statistically significant difference.

## Results

### Wortmannin blocks Ser473 phosphorylation of Akt and alters FOXP1 protein expression levels

EGF was used to activate the PI3K/Akt pathway, as MDA-MB-231 cells are known to exhibit basal pathway activity. Following a 5-min treatment with EGF, PI3K/Akt activity began to increase, with maximum PI3K/Akt activity exhibited 10 min after treatment (data not shown). After 15 min of exposure to EGF, the activity of the PI3K/Akt pathway decreased, therefore, a 10-min treatment was selected as the positive control.

**Figure 1:**
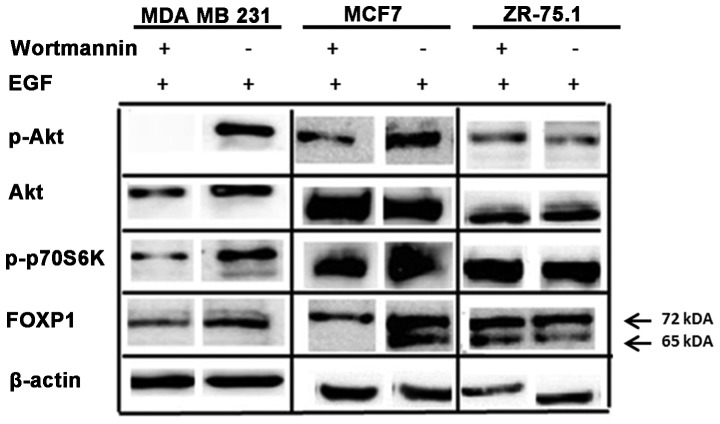
p-Akt, Akt, p-p70S6K and FOXP1 protein expression levels following wortmannin treatment in MCF7, ZR-75.1 and MDA-MB-231 cells. EGF, epidermal growth factor; p-Akt, phosphorylated Akt; p-p70S6K, phosphorylated p70S6 kinase; FOXP1, forkhead box P1.

Pharmacological inhibition of the PI3K/Akt signaling pathway was initially performed by treating the MCF7, ZR-75.1 and MDA-MB-231 cells with 100 nm wortmannin to elucidate the effect of Akt inhibition on FOXP1 protein expression levels. Following exposure to wortmannin, the MDA-MB-231 cells demonstrated decreased FOXP1 expression levels in parallel with a decrease in phosphorylated (p)-Akt (P<0.01) and p-p70S6K (P<0.05) expression. Conversely, control lysates exhibited basal p-Akt and FOXP1 expression levels. In the MCF7 cells, wortmannin treatment resulted in marginal p-Akt inhibition (P<0.01) and a concordant reduction in p-p70S6K (P<0.05) and FOXP1 (P<0.05) expression levels. Furthermore, wortmannin exhibited no inhibitory effect on p-Akt or FOXP1 expression in the ZR-75.1 cells (P>0.05). Three independent experiments were performed and representative blots are indicated in Fig. 1.

### Akt silencing and overexpression affects FOXP1 expression

**Figure 2:**
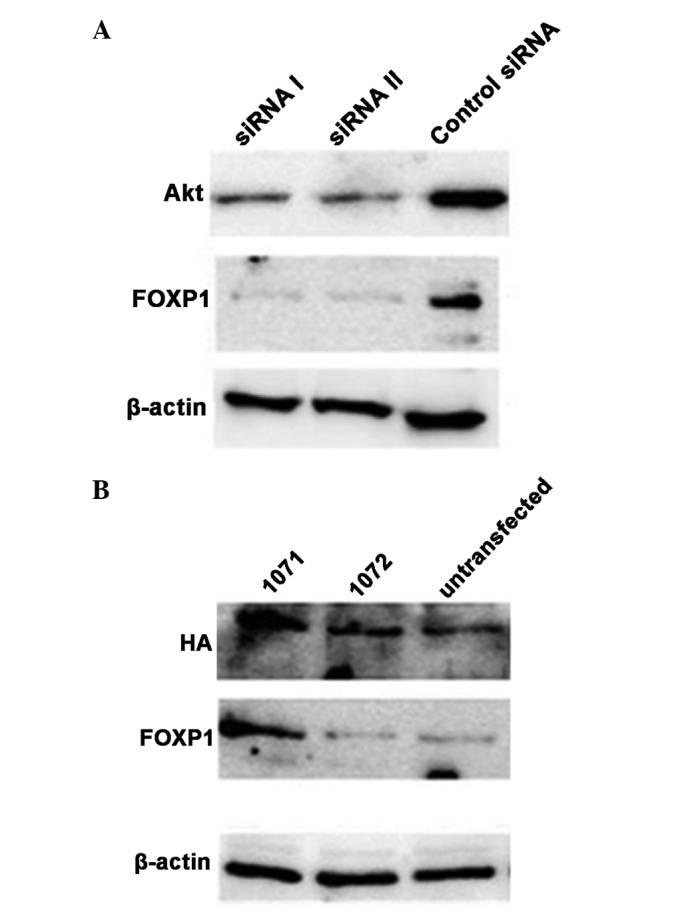
FOXP1 expression following siRNA silencing and overexpression of Akt. (A) Following siRNA-induced silencing of Akt, FOXP1 protein expression levels were decreased. (B) Following overexpression of Akt with HA-Myr-Akt1 (1071; kinase domain stable), a marginal increase in FOXP1 protein expression levels were observed compared with the untransfected control cells. However, following transfection with HA-Myr-Akt1 (1072; kinase domain mutated), FOXP1 expression did not change. siRNA, small interfering RNA; FOXP1, forkhead box P1; HA, hemagglutinin.

To further determine the effect of Akt inhibition on FOXP1 expression, the MDA-MB-231 cells were transfected with Akt siRNA and HA-Akt overexpression plasmids. To perform siRNA silencing, two Akt-targeted siRNAs and an untargeted control siRNA were used. Following siRNA transfection optimization, siRNA I and II provided effective silencing of Akt in MDA-MB-231 cells (P<0.001), however, the cells transfected with control siRNA did not inhibit Akt expression. Subsequent to the effective siRNA-induced silencing of Akt, the MDA-MB-231 cells demonstrated a decrease in FOXP1 protein expression levels (P<0.05; Fig. 2A). In a reciprocal experiment to upregulate Akt expression levels, the MDA-MB-231 cells were transfected with 1071 pBabe puroL and 1072 pBabe puroL overexpression plasmids. The highest FOXP1 expression levels were observed in the 1071 pBabe puroL transfected cells (P<0.05), where an increase in HA staining was also detected to confirm Akt1 overexpression; however, small expression bands were observed in the cells transfected with pBABE-puro empty vector (Fig. 2B). In addition, *Hin*dIII and *Bam*HI restriction endonucleases were used to ensure that no cross-contamination of the 1071 pBabe puroL and 1072 pBabe puroL AKt1 expression plasmids expression plasmids had occurred in the empty vector (data not shown). In contrast to the high FOXP1 expression levels observed in 1071 pBabe puroL-transfected cells (P<0.05), the 1072 pBabe puroL and empty vector-transfected cells demonstrated low FOXP1 expression levels (P>0.05).

### Rapamycin does not alter FOXP1 protein expression levels in MDA-MB-231 cells

**Figure 3:**
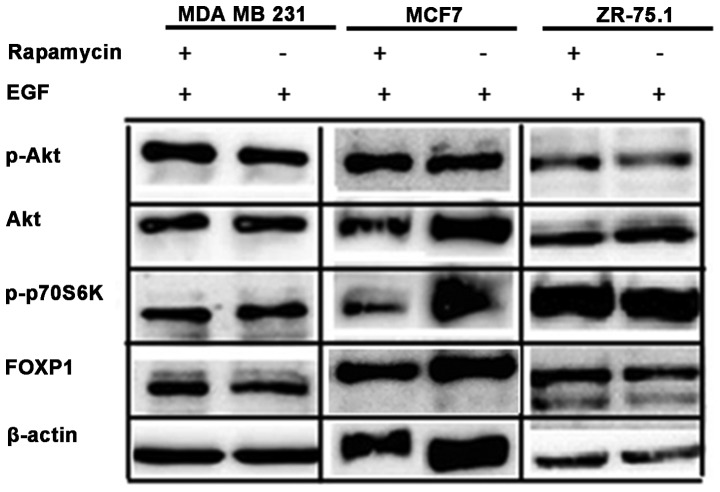
p-Akt, Akt, p-p70S6K and FOXP1 protein expression levels following rapamycin treatment in MCF7, ZR-75.1 and MDA-MB-231 cells. EGF, epidermal growth factor; p-Akt, phosphorylated Akt; p-p70S6K, phosphorylated p70S6 kinase; FOXP1, forkhead box P1.

The PI3K/Akt signaling pathway regulates FOXP1 protein expression levels; however, FOXP1 does not have any phosphorylation sites for Akt (11). Considering the proposal by Banham *et al* (11) that p70S6K has a recognition site for FOXP1, the present study investigated a possible association between p70S6K, a downstream target of Akt, and FOXP1 (11). The MCF7, ZR-75.1 and MDA-MB-231 cells were treated with rapamycin, a mammalian target of rapamycin (mTOR) inhibitor. In the rapamycin-sensitive MCF7 cells, p-p70S6K expression was decreased significantly (P<0.05) compared with the control cell lysate, however, p-p70S6K expression was unaltered in the MDA-MB-231 and ZR-75.1 cells. Furthermore, rapamycin treatment induced a significant decrease in FOXP1 expression (P<0.05) in addition to a decrease in p-p70S6K expression (P<0.05) in the MCF7 cells; however, the MDA-MB-231 and ZR-75.1 cells did not exhibit a decrease in these protein expressions following rapamycin treatment (Fig. 3).

### p70S6K silencing decreases FOXP1 expression in MDA-MB-231 cells

**Figure 4:**
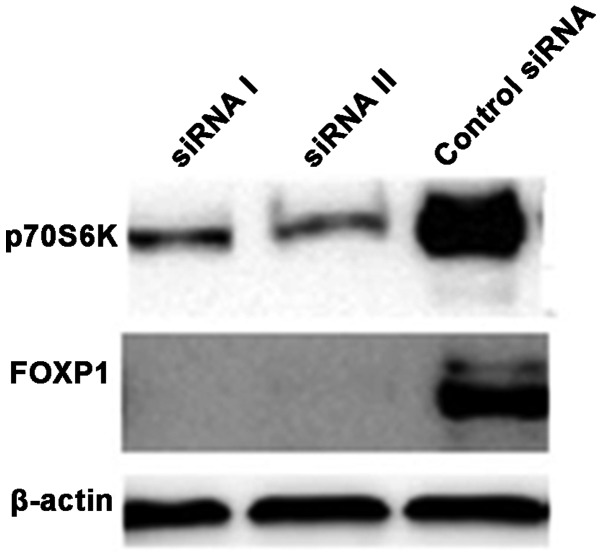
FOXP1 protein expression levels were significantly abrogated following siRNA-induced silencing of p70S6K in the MDA-MB-231 cells. siRNA, small interfering; p70S6K, p70S6 kinase; FOXP1, forkhead box P1.

To directly analyze the regulation of FOXP1 by p70S6K, the MDA-MB-231 cells were transfected with siRNA specifically targeting p70S6K. FOXP1 expression was reduced following p70S6K silencing with siRNA I (P<0.01) and II (P<0.01) (Fig. 4).

## Discussion

FOXP1, a winged-helix DNA-binding transcription factor, belongs to subfamily P of the forkhead box transcription factor family and has previously been associated with various types of cancer, with FOXP1 protein expression levels and different cellular localizations leading to distinct outcomes in different types of cancers. For instance, the high expression of smaller isoforms of FOXP1 (60–65 kDa) in diffuse large B-cell lymphoma (DLBCL) are associated with a poor prognosis, while recurrent chromosomal translocations effect *FOXP1* gene expression in B-cell non-Hodgkin lymphoma (12). FOXP1 is located on the tumor suppressor locus at chromosome 3p14.1, and trisomy of chromosome 3 has been associated with increased FOXP1 expression in mucosa-associated lymphoid tissue lymphoma. However, in breast cancer, FOXP1 acts as a tumor suppressor, as loss of nuclear FOXP1 expression and cytoplasmic mislocalization are associated with a poor prognosis, and increased immunoreactivity of nuclear FOXP1 is correlated with a low tumor grade and the status of the hormone receptors, ER-α and progesterone receptor (14–16). Consequently, identifying the regulatory mechanisms of FOXP1 expression and its functions is essential for the identification of novel therapeutic targets and the evaluation of the existing therapeutic inhibitors.

In the present study, it was demonstrated that the expression of FOXP1 is regulated by the PI3K/Akt signaling pathway. Decreased expression of smaller isoforms of the FOXP1 protein (60–65 kDa) occurred in the MDA-MB-231 cells following treatment with 100 nM wortmannin, while decreased expression of the larger FOXP1 isoforms (72-kDa) were observed in the MCF7 cells. Notably, the p-Akt and FOXP1 expression levels did not appear to be affected by wortmannin treatment in the ZR-75.1 cells (Fig. 1). To the best of our knowledge, no studies investigating wortmannin inhibition of PI3K/Akt signaling in ZR-75.1 cells have previously been conducted; however, the present study identified a correlation between FOXP1, and p-Akt and p-p70S6K expression levels in wortmannin-treated ZR-75.1 cells. As wortmannin did not cause inhibition of PI3K/Akt/p70S6K signaling, we hypothesized that no change in FOXP1 expression levels would occur. This mechanism may explain the steady state of FOXP1 expression in the ZR-75.1 cells. However, it was reported that wortmannin, which is primarily known as a PI3K inhibitor, also inhibits mTOR/p70S6K signaling in ZR-75.1 cells, possibly inhibiting p-p70S6K by blocking mTOR and thus abrogating the PI3K negative feedback loop (17–19), causing mitogen-activated protein kinase (MAPK) to be constitutively activated in human cancer. As MAPK is mutated in ZR-75.1 cells (20), wortmannin treatment may inhibit this constitutive activation, thus p-Akt and FOXP1 protein expression levels will remain constant.

In the present study, Akt silencing in the MDA-MB-231 cells resulted in a significant reduction in FOXP1 protein expression levels. By contrast, the 1071 pBabe puroL-transfected cells demonstrated a significant increase in FOXP1 expression compared with the 1072 pBabe puroL (catalytically inactive plasmid) and empty vector-transfected cells, as well as the untransfected cells (Fig. 2B). Normally, no HA-tagged primary antibody bands are present in empty vector-transfected and untransfected cells; however, bands similar to those for HA-tagged antibodies were identified in the empty vector-transfected and untransfected control cells of the present study. This may be due to the HA amino acid sequence cross-reacting with other proteins expressed in the MDA*-*MB-231 cells. This is supported by the results of a BLAST search performed on the HA amino acid sequence using the National Center for Biotechnology Information BLAST program (http://blast.ncbi.nlm.nih.gov/Blast.cgi), which identified numerous proteins with the same sequence.

Following the treatment of the MCF7 cells with rapamycin, FOXP1 expression was attenuated in parallel with decreased p-p70S6K expression levels. Additionally, a previous study identified that ZR-75.1 cells were not inhibited following treatment with 100 nm rapamycin for a time period of 20 min to 1 h (Fig. 3), however, ZR-75.1 cells did demonstrate sensitivity to rapamycin following a 4-h treatment (21). Furthermore, Sangai *et al* (20) demonstrated that after a 24-h exposure of the ZR-75.1 cells to rapamycin, the T389 phosphorylation of p70S6K was lost, whereas the present study determined that S371 phosphorylation of p70S6K occurred 20 min after the application of rapamycin. Thus, no sustainable changes in p-p70S6K and FOXP1 expression levels were detected in the ZR-75.1 cells. In addition, rapamycin did not appear to inhibit p70S6K phosphorylation in the MDA-MB-231 cells, which may explain the rapamycin-resistant property of this cell type (Fig. 4A) (20,21). Rapamycin typically inhibits the phosphorylation of p70S6K at nanomolar concentrations; for example, a previous study identified that a 20-nM dose of rapamycin inhibited the phosphorylation of the T389 residue of p70S6K in MDA-MB-231 cells (22). The present study evaluated S371 phosphorylation of p70S6K in MDA-MB-231 cells following treatment with 100 nM rapamycin. Although rapamycin is a highly specific inhibitor of mTOR, higher expression levels of phospholipase D (PHD), which functions in survival signaling and cell cycle progression and is essential for mTOR signaling, is associated with the development of rapamycin resistance in MDA-MB-231 cells via the phosphorylation of p70S6K and serum-induced Myc expression. By contrast, MCF7 cells were identified to be sensitive to rapamycin due to lower expression levels of PHD (23). Notably, p-Akt expression levels were increased following rapamycin treatment compared with expression levels in the EGF-treated MDA-MB-231 cells; in parallel with this increase in p-Akt expression, FOXP1 expression levels were marginally increased in the treated cells. It has previously been established that a negative feedback loop exists between p70S6K and PI3K, with p70S6K acting as a negative regulator of PI3K (24). However, if mTOR is inhibited by rapamycin treatment, this negative feedback mechanism is abrogated and PI3K is constitutively activated; in MDA-MB-231 cells this has been shown to result in increased S473 phosphorylation of Akt (24,25). By contrast, inhibition of mTOR complex 1 (C1) by everolimus treatment promoted the activation of the PI3K-dependent negative feedback loop, thus, activating the MAPK signaling pathway (19). Another previous study observed a subsequent increase in Akt phosphorylation 4 h after inhibition due to the function of rapamycin-insensitive companion of mammalian target of rapamycin (26).

p70S6K is a Ser/Thr kinase and a member of the AGC kinase family, and is also termed S6K1. p70S6K modulates the activity of numerous downstream proteins by regulating translation, and its expression levels are associated with cell size, regulation of protein synthesis, cell growth and cell proliferation. P70S6K is responsible for translational initiation via mTORC1, in particular regulatory-associated protein of mTOR, which specifically phosphorylates p70S6K, which in turn activates eukaryotic translation initiation factor 4B (eIF4B). eIF4B phosphorylation is a significant factor in the initiation of translation, for example, by enhancing the activation of eIF4A (27,28). Furthermore, p70S6K depletion in MDA-MB-231 cells and rapamycin treatment of MCF7 cells demonstrated that FOXP1 is regulated by p70S6K. This may be due to the translational regulation of FOXP1 by p70S6K, as decreased or depleted p70S6K may cause a decrease in FOXP1 protein translation. Additional studies are required to clarify this regulatory mechanism.

Numerous phase II clinical studies of agents aimed at targeting Akt in breast cancer have been conducted by the National Cancer Institute, the National Institutes of Health and other organizations. MK2206, an orally available allosteric inhibitor of Akt, is being used to treat patients with advanced breast cancer (http://clinicaltrials.gov/show/NCT01277757), and AZD5363, a novel orally available Akt inhibitor, is being used in the treatment of patients with phase I advanced or metastatic breast cancer and phase II ER-positive advanced or metastatic breast cancer (http://clinicaltrials.gov/show/NCT01625286). Additionally, a phase II study investigating mTOR targeting with rapamycin in Her2-positive metastatic breast cancer patients appears to have been aborted (http://clinicaltrials.gov/show/NCT00411788). However, the results of all studies aimed at inhibiting the PI3K/Akt/mTOR signaling pathway can be analyzed two ways, due to the dual roles of FOXP1 in cancer: While FOXP1 exhibits oncogenic functions in hepatocellular carcinoma, glioblastoma and DLBCL, it has also demonstrated suppressor roles in non-small cell lung, prostate and breast cancer (15,29,30). Furthermore, a number of correlation studies have demonstrated that the loss of FOXP1 protein expression is associated with a poor prognosis, however, the increased immunoreactivity of FOXP1 may predict a good response to tamoxifen treatment in breast cancer patients (16,31,32). FOXP1 also functions in the proliferation of MCF7 breast cancer cells. Thus, in conclusion, the PI3K/Akt signaling pathway should be considered as a target in the treatment of breast cancer, although additional studies are first required to determine the mechanism via which FOXP1 is regulated by the PI3K/Akt/p70S6K signaling pathway.
